# The Principles Involved in the Treatment of Diseased Conditions

**Published:** 1877-07

**Authors:** F. Peabody


### THE DENTAL REGISTER.

VOL. XXXI.] **JULY, 1877.** [NO. 7.

### THE PRINCIPLES INVOLVED IN THE TREATMENT OF DISEASED CONDITIONS.

BY F. PEABODY.

*Read before the Kentucky State Dental Association.*

The field this subject covers is so vast it will be impossible, in a paper as short as this will be, to do more than touch on some of the principal points. I do not expect to bring anything new before the association, and this paper is written rather for the purpose of gaining information from those, who I trust will discuss it, than with the idea that it will impart knowledge to any member of this body.

It is much to be regretted that so little is known of dental pathology by the profession at large; and that with from twenty to fifty or more members of the profession in almost every city, there can be found few, who have sufficient knowledge of this subject to take under their care and successfully treat any of the diseases common to the oral cavity, save those of the more ordinary character, which come under their observation every day. It is true that the community has not been taught to go to the dentist, in any trouble that cannot be traced directly to the teeth themselves; but it is the fault of the dentist, who has not prepared himself to undertake much more than the mechanical part, of what is termed operative dentistry, that this is so. When we, as a profession, have qualified ourselves, so that we can meet the general practitioner of medicine, or specialist in any of the branches of medicine, in an intelligent and scientific consultation in regard to disease, perhaps this may be changed.

The principles involved in the treatment of diseased conditions, rest on the four pillars on which the science of medicine is reared; without a knowledge of these we are powerless to treat disease intelligently. What is required then is, first, an intimate acquaintance with the organization of man, his anatomy, which teaches us how he is made and put together, the uses of the various parts, as well as their names, and their relations to other parts. Next a knowledge of physiology from which we learn the proper functions of the anatomical parts in life, their action and influence on each other part and on the whole. Then comes pathology, which is perverted physiology, or the natural actions or functions turned in a wrong or diseased direction. Lastly we have materia medica, with which to correct the pathological conditions, to restore abnormal to mormal functions, to relieve pain and give comfort and ease. Therapeutics which might be added to these though properly a branch of the science of medicine, which treats of the application of remedies, is in reality the grand application of the knowledge gained in all these directions. Having an acquaintance with these principles, we can act understandingly; without them, all efforts must be experimental and success accidental.

That which constitutes the cardinal principles of general medicine, constitutes also the principles of each of its specialities. No specialty of medicine, whether that of the dental surgeon, oculist, aurist, or any other, can be practiced in the most intelligent and thorough manner without an intimate knowledge of the whole of general principles. In dentistry, perhaps, it is not so essential to have a thorough knowledge of materia medica as in some of the other specialties; yet we frequently meet with cases where the first step towards the removal of local disease requires systemic treatment. We must have a knowledge of principles before we can apply remedies understandingly.

We cannot treat a disease until we recognize it, and having made a correct diagnosis, we must know its character, what its usual course is, what it will result in if left to itself, and what remedies will reach and correct it. In order to cure a disease we must strike to the bottom of it, and thoroughly eradicate from the system all trace of it. But while we are doing this, we must not forget that one of the best principles of treatment is based on the power of nature to restore itself; or that over-treatment often results in as much or more harm than under treatment. As the cause of disease is irritation, our first step should be to remove this, that is the first effort of nature; in order to do this she immediately establishes a condition of inflammation. Often she is incapable of succeeding in this unaided; then the skill of the physician is demanded. Having removed the cause, nature will take care of the result, for any system that has sufficient power to set up, and sufficient vitality to keep up a vigorous state of inflammation, in order to remove an irritation, has a corresponding power to restore itself when the conditions are changed, from abnormal to normal. Throughout the system there is constantly going on a process of waste and supply; and where there is a natural action, nature will bring to the parts requiring it, a sufficient amount of building material to restore them to health, if it is not beyond her power.

Let us look for a moment at the efforts of nature to remove an obstruction which is causing irritation. Did you ever put an obstruction over the opening to an ant hill? or see a hill of small red ants attacked by a large black one? What is the result? The sentinel on duty immediately sounds an alarm, a file of soldiers is sent out to remove the obstruction; if within their power they do it, and all goes on in the natural channels as before. If it is the big black ant that attacks the strong-hold, the file of soldiers bravely attack the enemy; they are not able to cope with him, reinforcements are sent for; he destroys many, undismayed they renew the attack, they swarm over the intruder, they surround him, on all sides they annoy him, they come bravely to the scene only to have their ranks depleted; they fall by scores only to have their places taken by others; they continue the attack until the ground covered by their dead and wounded is so heaped up that their own soldiers in turn become an obstruction, and the attack can no longer be carried on with any hope of success. Now bring your hand to the rescue; remove the enemy, clear away the slain; the coast clear, the remainder of the garrison go at once to work to restore the destroyed structure, grain by grain they replace the sand their house is built of, little by little, but slowly and steadily the work goes on; let them alone and in a short time the task is completed, and the edifice looks as full and perfect in its reconstruction as before it was invaded.

Take now (to apply this illustration) a necrosis of a portion of the superior maxillary. I speak of that because from its exposed position it is more subject to injury which may cause necrosis, and because its treatment pertains more directly to our profession.) The maxillaries have a greater degree of vitality than most of the bones, and consequently greater power to resist disease, and greater capability for reproduction, where a portion is lost from disease or otherwise. A part becomes necrosed, dead—that part is an irritant, it is an unnatural condition, the system immediately sends extra blood to the part to set up an inflammation to remove it. If the necrosed part becomes exfoliated nature, will succeed in casting it off, as it would a splinter from the hand. If it is not detached from the vital portion, more blood is sent, more inflammation is set up, regiment after regiment, squadron after squadron of corpuscles are sent to aid in expelling this extraneous object, which, from its change from a vital to a devitalized condition, has become a foreign intruder which stops up the channels and interferes with the natural course of the blood. Regiment after regiment and squadron after squadron fall in their efforts, they are powerless, nature converts her cohorts of blood-corpuscles into pus-corpusles, they undermine, they sap, they dissolve the surrounding tissues, they use all their efforts to hurl the enemy out. They fail, capillaries cease to perform their functions, they are filled with the dead and dying soldiers sent to the attack, a condition of sluggish action goes on and comparative stasis is the consequence. Then comes surgery, it comes with its instruments and removes the dead bone from the living, clears away all the debris of battle, depletes the engorged capillaries, opens the channels for the continuance of the natural flow, stimulates the absorbents to carry off what is not useful, what has been destroyed in removing the dead bone, and encourages the depleted capillaries to renewed exertion. The enemy is gone, the work of restoration must commence. Little by little the building material is brought. It is taken up and each atom is placed where it belongs, a cell is built here, another there, they reproduce, they grow, they increase, and in a short time all is completed, and, if the ravages of the disease have not been too great, the parts are restored, as far as possible, to their normal condition. I say as far as posssble, for I doubt if any reconstruction ever equals the original.

Nature is always at work restoring the waste and loss which are continual; we wish merely to aid her and give her a chance; she always does her work well; she wants some assistance at times, but she must not be killed by mistaken kindness.

The successful treatment of diseased conditions then, lies in a knowledge of the organization, a correct diagnosis of the disease and an intelligent and judicious administration and application of therapeutic agents, or the skillful use of surgical instruments, as the case may require. But aside from this knowledge many other things have to be taken into consideration; the general surroundings, the temperament, the question whether the disease is local or general, and if local whether it may not depend on some systemic cause, which must be removed before local treatment will be of any benefit; the condition of the blood, the habits of the patient, etc. Thus for instance, if we know, or learn upon inquiry that our patient has a hemorrhagic diathesis, we should be the better prepared to arrest any undue flow of blood in any operation that would cause bleeding. Locality, surroundings, and special conditions must be considered; results under one set of circumstances, or in one atmosphere, may not be repeated in another, any more than a successful operation in one mouth or in one tooth will necessarily be successful in all and a change in the form of disease demands a change of treatment. We know that gold, when properly used, will generally protect a tooth attacked by black or brown decay; while in teeth that are subject to white decay, tin, or some other filling is of a much more saving character, this is but a single example, and what you can count on with almost certainty in one case, may be of no advantage in another.

We, as a profession, have to deal more directly with the mouth and teeth, yet so intimately connected are the different organs, and so sympathetic with each other, that we frequently find troubles of the eye or ear entirely relieved by operations on the teeth. I have known instances where partial loss of sight has been restored by the removal of diseased teeth, and instances can be related by almost any dentist who has had any amount of practice, where deafness, either total or partial, has been cured by the removal of diseased teeth.

Our knowledge of the cause of disease is exceedingly limited, and a knowledge of etiology must necessarily be dependent on what information we may possess of pathology, just as that science is dependent on a knowledge of physiology. Perhaps less is known of the pathology of the mouth than of any other portion of the system; we know that the teeth decay, we know that the decays we find are of different characters, produced undoubtedly by different causes, but we have no positive knowledge of what causes these decays. We are often asked by our patients, "what makes the teeth decay?" How shall we answer them? I suppose generally the patient is informed that decay is caused by acids, and that these acids are produced by decomposition of food left between the teeth, or from the eructations of an acid stomach, or from acids taken into the mouth, and this is perhaps as good a general answer as can be given. Acids undoubtedly aid decay, and in some instances may be the cause of it, but unquestionably other causes are at work to produce the destruction of enamel and dentine, as we see it in the round holes in the crowns of the molars, irregularly shaped cavities in approximal surfaces, and elongated ones on the buccal portions of the necks of the teeth.

The human system in its full health and vigor is often capable of throwing off disease, the power of its vital force and energy is sufficient to prevent disease from obtaining a foothold; while under certain conditions of a perfectly physiological character, one portion may be so weakened (the greater force being called elsewhere) that it is not capable of resisting the insidious advances of disease.

Sometime ago I had for a patient, a young lady, just married; her teeth were well organized and she kept them cleansed with scrupulous care, I found it necessary to introduce some ten or twelve fillings to put her teeth in thorough order. Having given her minute instruction in regard to the care of her teeth, I spoke to her husband as to the proper diet, and explained to him the drain made by the system on the teeth in forming the osseous structure of the child, where the material was not found in sufficient quantities in the food. I requested the lady to let me see her in one year. It was eighteen months before I saw her again, in the mean time their union had been blessed with a child. I found in her mouth ten or twelve new cavities which had not existed when I saw her before; on questioning her she acknowledged that she had not paid much attention to the instructions I had given her husband.

It is said that a portion of the bone from the teeth, or rather that portion of bone-making material, which should have gone to the teeth for their proper support, is to a certain extent taken to supply the ossific wants of the embryonic child, and that the whole structure of the tooth in its impoverished condition is more susceptible of decay, or has less power to resist its encroachment in places favorable to its attack. How far this theory may be carried I am not prepared to say, my own opinion is that vitiated and changed secretions have quite as much to do with the decay of the teeth, under such circumstances, as any want of bone making material in the system. Be that as it may, and accepting the proposition as true, a knowledge of the requirements and course of nature under such circumstances must certainly be of great advantage and suggests the correctness of prophylactic treatment, which if it will accomplish the purposes, is certainly much better than remedial.

Experience often teaches us the best means with which to treat diseased conditions, and is of more value as we know, than information gained from books.

Life is too short, and the amount of knowledge to be acquired too great, for us to learn all that is necessary from our own observation, we have to accept much gathered from the observation and experience of others, particularly that which is generally accepted. In the treatment of diseases we frequently have to give remedies empirically, and in fact all medicines must have been used in that way at first, at least to some extent. We cannot in the treatment of new diseases, or in the administration of new remedies, go to work as in a problem in mathematics, and figure out with exactness what the results will be; therefore we must give remedies according to the best of our judgment, and watch and record the result. Having obtained that, we can then reason on their mode of action. Valuable remedies even to this day are given because they produce desired results, yet how these results are produced is not well understood. Experience then, teaches us how to use remedies, and from it we learn, that results reached in one instance (circumstances being the same) may reasonably be expected in another. While we sometimes have to use remedies, the action of which we cannot precisely explain, we often have to treat diseases the cause of which is hidden. We know in a painful tooth, where there is an exposed or congested pulp, if the pulp be pricked and a drop of blood taken from it, relief from the pain is often afforded. Why? Because the blood vessels in their congested condition are gorged, and in their enlargement consequent on that engorgement, pressure is caused on the nerve, it is irritated; the vessels being depleted by the removal of a drop of blood the nerve is relieved of its pressure, and the irritation is gone. If we fill a tooth over an exposed or nearly exposed pulp, where the metal comes in contact with the pulp or the thin stratum of bone over it, causing pressure, and some time after there is pain, or if a tooth be filled over a dead pulp, and there is pain, we know that by drilling into the root until the canal is reached we at once give relief. Why? Because the pulp either dead or dying is in a state of decomposition, and in decomposing generates gas; that gas swells trying to find an outlet, it causes pressure, therefore pain, if it were allowed to remain it would find an outlet through the alveolus, and establish an abscess; when we make an artificial opening the gas escapes, the pressure is removed and with it the pain. These are things we know, we know them from observation and experience, and from the teachings of others, they are matters that we can demonstrate. But who can give the cause of erosion of the teeth, a marked specimen of which I have with me, who can state positively what is the cause of what is known as tooth edgedness; can tell whether it be from a low state of circulation in the enamel, or from porosity, which admits of fluids being imbibed, or from some other cause? Who can explain the cause of the absorption of the roots of the permanent teeth, or the formation of secondary dentine in the pulp, or who knows the cause of the wasting of the sockets of the teeth, and the consequent loosening of the teeth, causing them to become useless in an otherwise apparently healthy condition? Theories of all sorts are advanced to explain these pathological conditions many of which must be taken "Cum grano salis" We do not know the cause of these troubles, consequently we cannot treat them as we could wish.

I have a patient in good health; the first and second molars on the inferior left side became loosened; the gums were apparently healthy; they became so annoying I had to remove them. The teeth were not decayed, the pulps were alive, and there was no absorption at the ends of the roots, the edges of the alveoli and septum were vital as far as I could distinguish. The same teeth on the right side are going in the same way. I have been treating them for two months in every way that I have been able to think of as likely to benefit them. I have stimulated the gums, explored the roots almost to their apexes, removed all traces of foreign accumulation, bathed them in elix. vit. to remove any thing that I might have overlooked, or to dissolve any dead portion of the alveolar process. I have done them no good. I did not expect to when I commenced. My patient will ultimately lose them, though she may keep them for years in their present condition; they cause her no pain and give her no uneasiness excepting becoming occasionally sore. I think she will lose all her teeth in the same way, they show indications of loosening; she keeps them perfectly clean, but in this case it is probably constitutional.

Of all the diseases that present themselves frequently for our treatment, perhaps there is none so common or so perplexing as that of recession of the gums, the loosening of the teeth, and the oozing of pus from around the necks of the teeth. I do not speak of those cases of inflammation and recession of the gums, which are evidently dependent on the encroachment of foreign substances, such as salivary calculus; these are more generally confined to the neighborhood of the mouths of the salivary ducts, including the lower front teeth and the superior molars. These cases are easily cured by removing the calculus, and if necessary stimulating the gums. But I allude to those cases where on one or more sides a broach can be introduced between the gum and tooth, sometimes entirely to the apex of the root, where there is no adhesion between the gum and root, and where the process and septum are to some extent absent. These cases are dependent, if not for their origin at least for their continuance on some other cause than the deposit of calculus. Now what is the cause? It has been the study of some of the more observant for years, and yet little is known as to what occasions this disease, or what is the best and most successful method of treatment. Is it owing to a stasis of circulation from whatever cause, which occasions the breaking down and absorption of the edges of the septum and process; to some chemical action, to some foreign substance entering under the free margin of the gum and keeping up a constant irritation; to the action of mercury or some other medicine; to some abnormal growth; some animalcule; or to some constitutional defect? My own experience has been in most cases that there is a necrosis of the edge of the alveolus. Dr. Riggs of Hartford, has given us more light on this subject than any other member of the profession, and I have understood that he succeeds in curing a large percentage of the cases that come under his care, and I also heard a prominent member of the profession say he "would about as soon die as go through his hands" his treatment is so severe.

A few months ago I had an interesting case of this character. The patient was a young lady who prized her teeth above every thing else; the tooth, a beautiful lateral incisor, stood some what apart from the central, and pressed closely against the canine, it was without blemish; it was not much loosened but there was the dark blue line at the edge of the gum which indicates trouble beneath. I found the alveolus, between the central and lateral dead for some distance up the root. I cut it away as well as I could at the first sitting, and in a day or two the dark line had disappeared and the gums had assumed a more normal appearance; but the trouble was not exterminated for I could still detect necrosed bone. I saw the patient twice a week, and each time I cut away more bone which I had neglected to remove at the previous sitting, until I had traced the necrosis some distance under the central incisor. I finally succeeded in removing it all, at least I could detect no more, and granulation commenced satisfactorily. Now the question arises have I succeeded in curing the case? and if so is the cure permanent? If the cause of the disease was the dead edges of the alveolus, and if I have succeeded in removing it all, undoubtedly the lost bone will be restored and no more trouble will arise, unless from a new attack of what caused the trouble before. Up to the present time it is doing well, some tenderness but no inflammation or oozing of pus. I believe she will have no further difficulty; but what caused the disease? No other tooth was in any way affected, and there was no indication of the trouble at any point on the gums, save the tooth that I treated. Had not the young lady possessed a courage almost spartan in its character, she could not, and would not have submitted to the operation, for it was exceedingly painful, few would have been willing to endure it, and on that account I do not consider it practicable. Aromatic Sulphuric Acid, it is stated, will dissolve the dead bone and destroy abnormal action without injury to living tissue. I have never succeeded in curing an aggravated case of this disease by this means, though some cases have been greatly benefited by an application of it.

I could give other cases and bring forward many other problems for solution, which would be of undoubted interest, but this paper has already exceeded the limits intended. On closing I would only say we cannot over estimate the importance of careful study, close observation, noting the result of our experience and comparing it with that of others, that thus we may acquire the means of combating diseased conditions, but this skill is not to be gained in a day. Nor can any degree of perfection be arrived at without labor. As the microscopic cell, by its multiplication and enlargement, forms the several parts of the human body; and these, by their harmony and solidarity, form the structure man, so do the atoms of knowledge which we gather here and there, by observation and experience, and store away in the recesses of the brain, each a power in itself, yet dependent on every other, and strengthened by every additional atom, unite to form that living body of science which is the source of all our art. From it as from a fountain, flows that light which guides us in our efforts to contend with disease and to turn pathological into physiological conditions.

